# Odor Sampling: Techniques and Strategies for the Estimation of Odor Emission Rates from Different Source Types

**DOI:** 10.3390/s130100938

**Published:** 2013-01-15

**Authors:** Laura Capelli, Selena Sironi, Renato Del Rosso

**Affiliations:** Politecnico di Milano, Department of Chemistry, Materials and Chemical Engineering “Giulio Natta”, Piazza Leonardo da Vinci 32, 20133 Milano, Italy; E-Mails: selena.sironi@polimi.it (S.S.); renato.delrosso@polimi.it (R.D.R.)

## Abstract

Sampling is one of the main issues pertaining to odor characterization and measurement. The aim of sampling is to obtain representative information on the typical characteristics of an odor source by means of the collection of a suitable volume fraction of the effluent. The most important information about an emission source for odor impact assessment is the so-called Odor Emission Rate (OER), which represents the quantity of odor emitted per unit of time, and is expressed in odor units per second (ou·s^−1^). This paper reviews the different odor sampling strategies adopted depending on source type. The review includes an overview of odor sampling regulations and a detailed discussion of the equipment to be used as well as the mathematical considerations to be applied to obtain the OER in relation to the sampled source typology.

## 1. Introduction

Odor characterization and measurement have become very important environmental issues, due to the general public's growing awareness of the environment and the impact of air quality on health and well-being [1]. In recent decades, several techniques have been studied and developed for the characterization of environmental odors.

The oldest and most consolidated method of odor characterization involves the use of analytical techniques for the identification and quantification of odorants, such as gas-chromatography coupled with mass spectrometry (indirect methods). It is worth to mention that in recent years, several on-line methods based on mass spectrometry (MS), such as Membrane Inlet MS (MIMS), Selected Ion Flow Tube MS (SIFT-MS), Proton-Transfer-Reaction MS (PTR-MS), or Proton-Transfer-Reaction Time-Of-Flight MS (PTR-TOF-MS), have been studied and applied for the assessment of environmental odorants, especially in the agricultural sector [2–6]. However, chemical analysis can turn out to be highly complex and not always effective. This is particularly true in the characterization of complex odors, for which it is difficult to relate the sensation provoked by an odorous mixture in humans to its chemical composition [7,8], mostly due to the highly complicated effects of odorant mixing [9–11]. For these reasons, sensorial techniques, such as dynamic olfactometry [12], which allow odors to be characterized as a result of their direct effects on a panel of qualified examiners (direct methods), are being used increasingly frequently for odor impact assessment purposes. Dynamic olfactometry has the drawback of being affected by the variability of human olfaction, which has been only partially overcome by recent standardization [13,14], and of allowing to odors to be quantified solely in terms of intensity or concentration, without providing any information on odor quality. Lastly, some recent studies have focused on the possibility of monitoring environmental odors by using senso-instrumental techniques [15,16], *i.e.*, electronic noses artificially performing the functions of human olfaction, whereby an array of partially-selective sensors yields a characteristic pattern of the air analyzed, which is subsequently classified based on a reference database built up by the instrument in a prior training phase [17]. Environmental applications of electronic noses include both source characterization [18] and ambient air monitoring at receptors [19,20]. However, those applications are still limited by certain instrumental problems, most of which are associated with sensor sensitivity to variable environmental conditions (e.g., temperature and humidity) [21] as well as their stability over time [22].

Regardless of the measurement technique adopted (dynamic olfactometry, chemical analysis or electronic nose), the quality of the results obtained is heavily dependent on appropriate sampling, which is one of the main issues relating to odor characterization and measurement.

This paper does not explore characterization and measurement methods in any great detail as these were only recently extensively reviewed by Brattoli *et al.*[23]. However, it does focus on sampling and discusses current best practices as well as highlighting aspects that remain problematic and thus warrant further research.

The aim of sampling is to obtain representative information on the typical characteristics of odor sources by means of the collection of a suitable volume fraction of the effluent.

Typical characteristics of odor sources include:

- temporal trend of the emission, including emission peaks;
- transfer modalities of odorous substances from the source to the atmosphere;
- geometrical source configuration, *i.e.*, point, surface or volume source.

The sampled operating conditions, and the number and duration of samplings should be selected to allow comprehensive evaluation of the odor impact associated with the monitored source.

Two different types of sampling methods exist: dynamic and static sampling [12]. Dynamic sampling involves the air flow to be analyzed being ducted directly from the source to the measurement device. The duct may be heated in order to minimize adsorption or condensation phenomena on the duct walls. In this case, the measurement device must be installed in close proximity to the odor source, *in situ* or in a mobile laboratory (e.g., a van) in order to minimize pressure drops. If there is a risk of particulate precipitation in the device, the sampled flow should be filtered through a glass-fiber filter before it enters the device. In order to avoid condensation phenomena, the filter should be heated to the sample temperature.

Static sampling involves the sample being enclosed in a suitable container (canister or bag) which is connected to the measurement device at a later stage. Condensation and adsorption phenomena must be avoided during the storage phase. The sampling equipment should be odorless and minimize any interaction between the sampled gas and the surfaces it comes into contact with. Consequently, both the surfaces and storage times should be minimized.

Dynamic sampling has the advantage of minimizing the risk of sample modifications because of adsorption on the sampling equipment or chemical reactions between the compounds contained in the sampled gas which might occur while the sample is being stored. Olfactometric analyses require a panel of carefully-selected human examiners but dynamic sampling has the drawback of being very expensive as it requires the examiners to be taken to the site. Moreover, the presence of the panel on site may negatively affect its responses, due both to consciousness of the sample's provenance and the possible presence of background odors.

For the abovementioned reasons, static sampling is by far the most widely used methodology for olfactometric measurements as well as the only sampling methodology currently the focus of research and standardization.

This paper focuses on odor sampling for olfactometric analyses and thus aims to review the most important aspects of static sampling, including the techniques and the strategies that need to be applied to estimate odor emission rates from different source types. In this context, the paper also gives an overview of the issues covered by current odor sampling legislation, thereby highlighting those issues that have not been tackled sufficiently and remain problematic, and widening the investigations of present and future research activities into the subject.

## 2. Regulation on Odor Sampling

As previously mentioned, sampling is a critical issue as far as odor measurement is concerned. Nonetheless, no specific odor sampling guidelines have existed until now.

The European Standard EN 13725:2003 on olfactometry “Air quality—Determination of odor concentration by dynamic olfactometry” [12] includes a section on sampling (Sections 6 and 7, Annex J), even though in the introduction to the document the authors note that: “Improvements in sampling may be the subject of a future revision of this European Standard”.

More specifically, Sections 6.2 and 6.3 of EN 13725:2003 deal with sampling materials, whereas Section 7 defines the possible sampling methods (dynamic *vs.* static) and the procedures to be adopted to ensure the olfactory characteristics of the sample are kept as constant as possible between sampling and analysis, *i.e.*, sample pre-dilution, transport and storage. Annex J “Sampling strategies” of the EN deals with the techniques to be used for sampling depending on the monitored source type. Nonetheless, the indications given are not exhaustive, especially as far as “diffuse sources” are concerned, thus leaving many degrees of freedom in the choice of sampling procedures and equipment. This is one of the main criticisms of this standard and of odor measurement in general, as it has led to the development of a considerable number of different odor sampling methods and devices all over the world [24].

Given these difficulties and the importance of the problem, the German VDI (Verein Deutscher Ingenieure—Association of German Engineers), which has always been very active in this field (EN 13725:2003 was derived almost entirely from the German VDI 3881 [25]), recently published a specific odor sampling guideline. VDI 3880 [26] discusses all aspects of odor sampling in greater detail, particularly aspects partly overlooked in the EN. It provides specific information and examples of the procedures and equipment to be used for odor sampling from different source types.

The paragraphs that follow, which deal with sampling and estimation of the odor emission rate, cover both the EN 13725 and the VDI 3880, summarizing the overall aspects and the most important points of both documents, thereby highlighting problematic areas currently under study and which still require standardization.

## 3. Planning of Sample Collection and Analysis

To achieve an exhaustive characterization of the emissions of any industrial plant, a monitoring plan must be drawn up in order to gather as much significant information as possible about the plant's odor impact from each single analysis, thus avoiding measurement errors or pointless replication.

Sampling and analysis must be conducted with the aim of obtaining results representative of the monitored plant's emissivity. Consequently, it is important to gather sufficient information about the plant and its emission sources before sampling commences. An in-depth knowledge and analysis of the production cycle and of all the plant's other activities are fundamental to identifying its main odor sources.

It may be important to know the chemical composition of the emissions, and to gather information regarding the possible presence of toxic compounds in the sampled effluents. This is important to ensure the safety of the sampling operator and the examiners performing the olfactometric analysis.

It may therefore be useful to have an idea of the chemical compounds that might be contained in a sample prior to sensorial assessment. This can be done by either studying the plant's production cycle, or, if necessary, running a detailed chemical characterization of the emission to be sampled.

An extraction phase prior to the analysis phase is required to run a chemical analysis of a gaseous emission as the compounds to be detected are often typically at low concentration levels (often in the range of ppm or even ppb). Extraction techniques include cryogenic pre-concentration, liquid-liquid extraction, solid phase extraction, purge and trap, and solid phase microextraction (SPME). These techniques allow the pre-concentration of the compounds to be analyzed at a stationary phase and then their subsequent thermal desorption or elution with a solvent [27,28].

Precise identification of sampling points is also very important from a logistical point of view to allow sampling operations to be organized. In certain cases, it may be necessary to take specific precautions to either simplify sample collection or even make it achievable (e.g., creation of sample plugs, arrangement of safe methods of getting to hard-to-reach sampling points, use of specific sampling equipment, *etc.*).

Given these considerations, an accurate preliminary survey of the plant to be monitored and the drafting of a detailed report spanning all the identified sampling points, as well as notes regarding sampling modalities are essential to the effective planning of any olfactometric monitoring project.

## 4. General Requirements for Sampling

General requirements for sampling are specified in the EN 13725 and the VDI 3880. Just some of the main aspects of sampling will be discussed in the paragraphs below with certain additional considerations included to highlight critical aspects of, and the differences between, the two abovementioned regulations.

### 4.1. Sampling Materials

Generally speaking, sampling materials, *i.e.*, any material that may come into contact with the gas sample from the moment sampling occurs to analysis in the laboratory, should be chosen to avoid any contamination that might alter the sample's olfactory properties.

For this reason, sampling materials for odor measurement should meet the following requirements:

- Inertia: materials should minimize the possibility of interactions with the sampled gas. Inert materials are: polytetrafluoroethylene (PTFE, Teflon™), copolymer of tetrafluoroethylene and hexafluoropropylene (FEP), polyethyleneterephtalate (PET, Nalophan™), glass (drawback: fragility), steel (advantage: high mechanical and thermal stability; drawback: not always chemically inert, condensations or other depositions cannot be visually verified);
- Smooth surface;
- Absence of odor: they should not add odor to the sampled gas;
- Low permeability: they should avoid sample losses by diffusion or incoming of outside air.

One very important issue with regard to sampling materials is the choice of the material used to make the sampling bags. Any of the above-listed soft materials (*i.e.*, FEP, PET and PTFE) are suitable for this purpose.

Possible new materials for use in sampling bags must be tested and proven to be odorless. Other tests should concern the permeability of the material to odorous compounds. In point of fact, it is important to be aware of any diffusion processes through the sampling bags that might possibly lead to a decrease in odor concentration over time.

Recent studies have shown that some of the most widely available materials that are most frequently used for odor sampling bags, such as Nalophan™, Teflon™ or Tedlar™, have non-negligible diffusion coefficients with respect to specific odorous substances [29–31], especially if the latter are small molecules or soluble in water, as is the case with ammonia (NH_3_), hydrogen sulphide (H_2_S) or formaldehyde (CH_2_O), resulting in a decrease of the measured odor concentration over time [32]. The diffusion of specific molecules may depend on intrinsic factors, such as bag thickness, as well as extrinsic factors, such as the temperature and humidity at which the sample is stored. This phenomenon must be taken into account for sample storage, especially when dealing with emissions containing considerable amounts of the abovementioned compounds, such as emissions from landfills [33,34], wastewater treatment plants [35,36], livestock [37] and foundries [38,39]. In such cases, it may be necessary to consider possible counter-actions such as adopting special sampling strategies or materials [40], or reducing storage time. Methane (CH_4_) has been shown to diffuse through Nalophan™ bags. This might be a problem for the chemical analysis of landfill gas, the main constituent of which is methane [41]. However, this shouldn't have a significant effect on olfactometric analyses since methane is non-odorous.

Another important aspect relating to sampling materials is the experimental bias associated with air (or gas) sampling [42,43], due to the release over time of odorous substances from the polymeric film used to make sampling bag (monomers or other by-products for the most part). This effect was studied in particular for Tedlar™ bags, which typically tend to have a characteristic phenol background odor [44]. However, background odor was also found to be a problem for Nalophan™ bags [45]. This issue can, however, be overcome by sample conditioning, *i.e.*, by filling and then evacuating the bag with the gas to be sampled at least once, or by flushing it with the sample flow for an appropriate amount of time before making the final sample collection which will remove the odorous molecules accumulated inside the bag [12].

### 4.2. Sample Collection and Storage

Sample collection duration must be long enough to guarantee the sample is representative of the monitored emission. That said, short sample collection times may also reduce the risk of contamination during sampling. EN 13725 doesn't give any instructions in this regard whereas the minimum sampling duration per sample established by VDI 3880 is 30 minutes.

The time that elapses between the collection of the sample and its analysis (*i.e.*, the sample storage time) should be minimized to reduce the risk of sample modification during storage. The EN 13725 sets maximum storage time at 30 hours, whereas the recent VDI 3880 reduces it to just 6 hours.

Given the abovementioned studies which demonstrate that odorous substances can diffuse through the sampling bags, the storage time of 30 hours indicated by EN 13725, may be too long in order to guarantee sample olfactory properties are preserved. Nonetheless, a shorter storage interval, such as the 6 hours indicated by the German VDI 3880, may be too short to allow the analysis of samples collected from facilities plants located at a considerable distance from the laboratory. In such cases, mobile labs would be required and this would raise the costs involved considerably.

## 5. Sampling Strategies

### 5.1. General Principles

In odor measurement, measuring odor concentration alone is not sufficient in itself as the air flow associated with the monitored odor source must also be accounted for, as, in most instances, these parameters are related to each other. The basic parameter is the Odor Emission Rate (OER), which is expressed in odor units per second (ou_E_·s^−1^), and is obtained as the product of odor concentration and air flow associated with the source. EN 13725 states that volumetric air flow should be evaluated in normal conditions for olfactometry: 20 °C and 101.3 kPa on a wet basis.

The method adopted for the estimation of the OER from an odor source depends on source type. Consequently, different sampling strategies should be adopted to suit the source to be monitored [46,47].

### 5.2. Point Sources

In the case of point sources, odor is emitted from a single point, generally in a controlled manner through a stack, and sampling involves the withdrawal of a fraction of the conveyed air flow. The emitted air flow can be calculated by measuring air velocity and the cross-section of the duct. The OER is then obtained as follows:

(1) $$\text{OER}=Q_{\text{air}}\cdot c_{\text{od}}$$

where *OER* is the Odor Emission Rate (ou_E_·s^−1^); *Q_air_* the effluent volumetric air flow (m^3^·s^−1^) and *c_od_* the measured odor concentration (ou_E_·m^−3^). If the gas to be sampled is pressurized, withdrawal may be carried out by inserting the sampling bag directly into the duct.

**Figure 1.**
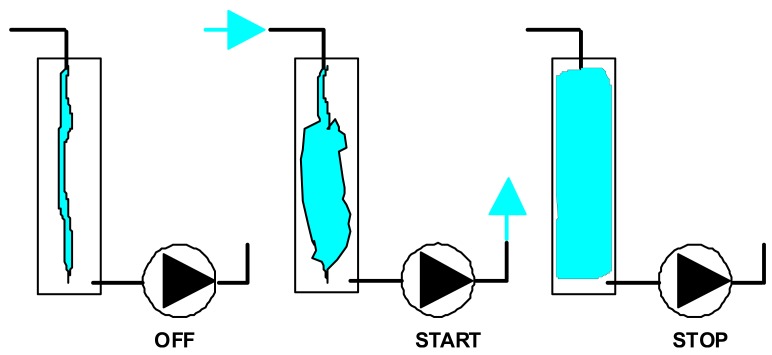
Scheme of sampling by means of vacuum pump.

In most cases, effluent pressure is not sufficient to inflate the sampling bag, and withdrawal therefore requires the creation of a depression. For this purpose, the sampling bag is inserted into a suitable container, then the air inside the container is sucked out by a pump. Because of the vacuum depression (vacuum?) created inside the container, the gas to be sampled is sucked into the sampling bag in an indirect manner, thus preventing the sampled gas coming into contact with the pump (Figure 1).

### 5.3. Volume Sources

Volume sources are typically buildings from which odors emerge either intentionally through naturally ventilated ducts, or unintentionally through doors, windows or other openings. Characterizing emissions from such sources is challenging as it is difficult to measure a representative odor concentration. It is also often simply not possible to define a precise air flow and thus specific techniques are required to estimate the OER. One possibility is to collect a sample of the ambient odor inside the building using the same depression pump as for point source sampling (assuming the odor concentration to be well mixed within the building) and then to estimate the air flow through the openings (dispersion factor) [48]. Estimation of the air flow from volume sources might be based on the use of suitable tracer gases, such as SF_6_ [49,50] or CF_4_ [51].

### 5.4. Surface Sources

In the case of surface sources, emissions typically come from extended solid or liquid surfaces. Two different kinds of surface sources may be distinguished [46]:

- Active surface sources, *i.e.*, sources having an outward air flow (e.g., biofilters or aerated heaps);
- Passive surface sources: *i.e.*, sources without outward air flow, the mass flow from the solid or liquid surface to the air (volatilization) is due to phenomena such as equilibrium or convection (natural or forced). Examples are landfill surfaces and wastewater treatment tanks.

The distinction between the two kinds of sources may not always be obvious. For instance, in the case of biological oxidation tanks for wastewater treatment, which do have an outward flow, even though it may be very low. In such cases, it is possible to establish a volumetric air flow limit to distinguish between active and passive sources. The VDI 3880 [26] sets a specific flux limit of 50 m^3^·h^−1^·m^−2^.

#### 5.4.1. Active Surface Sources

**Figure 2.**
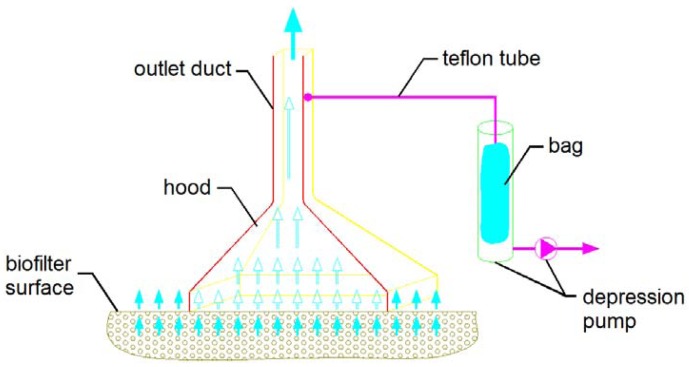
Scheme of sampling from an active surface source.

In the case of active surface sources, sampling is performed by means of a “static” hood that isolates a part of the emitting surface thus channeling the outward air flow into the hood outlet duct where the sample is collected using the same method as for point sources (Figure 2).

In general, a static hood consists of two main parts: a cone or pyramid frustum with a known base area (e.g., 1 m^2^), and a stack, generally cylindrical and with a diameter of 10–20 cm, on top of it. One or more openings are made in the stack to allow sample collection and the measurement of physical parameters, such as temperature, relative humidity or velocity. The sampling hood must be made using permitted materials, *i.e.*, odorless and inert.

For sampling to take place, the hood must be positioned on the emitting surface to isolate the sampling point from the outside atmosphere thus preventing the sampled gas being diluted by wind.

To obtain representative data of the entire source, samples must be collected from different points which should be uniformly distributed over the emitting surface. Generally speaking, the surface portion sampled should be about 1% of the total [52]. For example, if using a static hood with a base area of 1 m^2^ on a biofilter of 500 m^2^, five samples should be taken from five different points uniformly distributed over the whole surface.

It is important to verify the uniformity of the emitted flow throughout the surface in order to define the average emitted odor concentration, *i.e.*, the average value that, when multiplied by the effluent flow, gives the OER.

Two different cases can be distinguished:

- active surface sources with homogeneous flow distribution;
- active surface sources with non-homogeneous flow distribution.

Active surface sources should be considered as having a homogeneous flow distribution if the differences between the measured effluent velocities on monitored surface portions are below a defined factor. VDI 3880 [26], for instance, suggests a factor of 2.

In the case of homogeneous flow distribution, the average odor concentration is obtained as the geometric mean of the odor concentration values of the collected samples, in line with the following equation:

(2) $${\bar{c}}_{od}=\sqrt[{n}]{\prod_{i=1}^{n}c_{i}}$$

where *c̄_od_* is the average odor concentration and *c_i_* the odor concentration measured on the *i*-th surface portion, both expressed in ou_E_·m^−3^.

However, in the case of non-homogeneous flow distribution (the differences between the velocities measured on the different surface portions are higher than the fixed factor, e.g., 2), the average odor concentration should be weighted by effluent velocity, as per the following equation:

(3) $${\bar{c}}_{od}=\frac{\sqrt[{n}]{\overset{n}{\underset{i=1}{\text{∏}}}\left(c_{i}\cdot v_{i}\right)}}{\overset{n}{\underset{i=1}{\text{∑}}}v_{i}}$$

where *c̄_od_* is the average odor concentration (ou_E_·m^−3^); *c_i_* the odor concentration measured on the *i*-th surface portion (ou_E_·m^−3^) and *v_i_* the effluent velocity measured on the *i*-th surface portion (m·s^−1^).

The aforementioned equation is valid if the surface portions considered have the same area, otherwise the average odor concentration should be further weighted as follows:

(4) $${\bar{c}}_{od}=\frac{\sqrt[{n}]{\overset{n}{\underset{i=1}{\text{∏}}}\left(c_{i}\cdot v_{i}\cdot S_{i}\right)}}{\overset{n}{\underset{i=1}{\text{∑}}}\left(v_{i}\cdot S_{i}\right)}$$

where *S_i_* is the area of the *i*-th surface portion (m^2^).

#### 5.4.2. Passive Surface Sources

In the case of passive surface sources, OER estimation is a rather complicated process as it is difficult to measure a representative odor concentration, and, most importantly of all, to determine a well-defined air flow rate.

**Figure 3.**
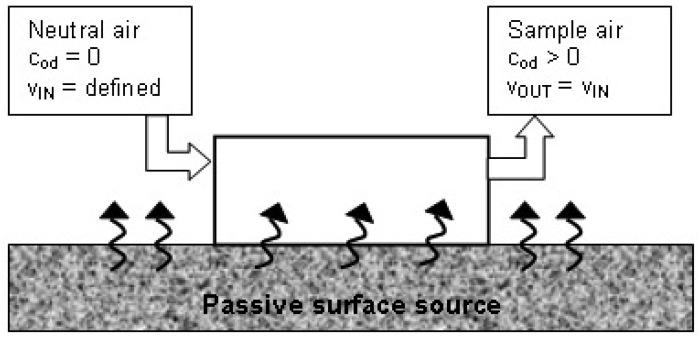
Principle of hood sampling on a passive surface source.

Generally speaking, the estimation of emission rate values from passive area sources may be performed using either one of two different approaches [53]:

- indirect measurements using micrometeorological methods, where emission rates are derived from simultaneous measurements of wind velocities and concentrations across the plume profile downwind of the source;
- direct measurements using an enclosure of some sort, *i.e.*, so-called “hood methods”. In this case, emission rates are derived from the data regarding the concentration of the compounds of interest measured in the samples collected at the outlet of the sampling device combined with the dimensions of the device and the operating conditions (Figure 3).

Indirect techniques, such as micrometeorology, do not interfere with the emission process because a sampling device is not used. However, such techniques are not practical for odor assessments because of the large number of samples required to characterize the emission under consideration. For this reason, hood methods are by far the most widely-used techniques for the evaluation of emission rates from passive area sources, and will be covered more extensively in this section.

Various sampling devices have been designed and tested for the collection of samples from a range of passive surface sources, and have also been recently exhaustively reviewed by Hudson *et al.*[24]. They include “static” and “dynamic” hoods which differ from each other principally with regard to the directionality and flow rate of the carrier gas insufflated inside the hood [54].

Older studies deal with the use of “static” hoods, *i.e.*, flux chambers [55,56], in which mixing between the carrier gas and the emission to be sampled is non-directional, and the carrier gas is generally supplied at a flow rate of between 5 and 24 L/min. One drawback associated with the use of “static” hoods for odor sampling on passive surface sources is that they may affect the gas exchange, especially due to pressure variations inside the hood, and thereby alter the sample representativeness [57]. Other studies prove the flux chamber techniques to be associated with critical errors due to compound instabilities in the chamber [58].

In recent years, “dynamic” hoods, *i.e.*, “wind tunnels”, in which the carrier gas is introduced in a directional way to simulate the action of the wind on the sampled surface, have almost completely replaced flux chambers for passive source odor sampling because they allow better characterization of the effects of natural ventilation on odor emissions [53,54].

Wind tunnels can be operated at different neutral air flow rates: the use of higher flow rates, which create air velocities of between 0.3 and 1.0 m/s inside the hood, has the advantage of simulating typical environmental conditions that may occur on the surface to be sampled [54,59,60]. The drawback of this choice is that such high air flow rates may dilute the emission to sampled too much, thereby making it difficult to measure odor concentration by dynamic olfactometry. For this reason, low-speed wind tunnels, working with air velocities of few centimeters per second inside the hood (e.g., 1–10 cm/s), are generally preferred [61,62].

As already mentioned earlier, in spite of their differences, all these devices are based on the same principle: to isolate a portion of the emitting surface by means of a hood, to insufflate a neutral (*i.e.*, odorless) air stream and finally to measure the odor concentration at the hood outlet.

Based on this principle, OER estimation requires the calculation of another significant parameter, *i.e.*, the Specific Odor Emission Rate (SOER), expressed in odor units emitted per surface and time unit (ou_E_·m^−2^·s^−1^), as per the following equation:

(5) $$\text{SOER}=\frac{Q_{\text{air}}\cdot c_{od}}{A_{\text{base}}}$$

where *SOER SOER* is the Specific Odor Emission Rate (ou_E_·m^−2^·s^−1^); *Q_air_* the air flow rate inside the hood (m^3^·s^−1^); *c_od_* the measured odor concentration (ou_E_·m^−3^) and *A_base_* the base area of the hood (m^2^).

The OER is then calculated by multiplying the SOER by the emitting surface of the source:

(6) $$\text{OER}=\text{SOER}\cdot A_{em}$$

where *OER* is the Odor Emission Rate (ou_E_·s^−1^); *SOER* the Specific Odor Emission Rate (ou_E_·m^−2^·s^−1^) and *A_em_* the emitting surface of the considered source (m^2^).

In order to obtain results representative of the real emission scenario, special care must be taken to prevent the sampling hood, which covers and isolates a portion of the emitting surface, altering the emissivity of that portion of the surface. For instance, a pressure variation inside the hood might suppress or encourage the volatilization of odorous substances. For this reason, it is important to leave sufficient time between the positioning of the hood on the sampling surface and sample collection. How much time generally depends on the characteristics of the hood (e.g., geometry and dimensions).

In most cases, sampling on passive surface sources is performed using wind tunnels. This kind of hood is designed to simulate parallel flux atmospheric conditions without vertical mixing: a horizontal neutral air flow causes volatilization of odorous compounds from the sampled surface, thus resulting in odor emission. In order to extrapolate the data obtained during sampling to real environmental conditions, some knowledge of the physical phenomena involved in the volatilization of the odorous compounds from the surface to the sampled air is required.

For liquid surfaces, this phenomenon of mass transfer to a gas phase, which is known as forced convection, can be successfully described by means of the Prandtl boundary layer theory [63]. According to the latter, the mass transfer coefficient relevant to a compound can be expressed with the following equation:

(7) KC=0,664DilRe1/2Sc1/3

where *K_C_* is the mass transfer coefficient (m·s^−1^); *D_i_* the molecular diffusivity of *i* in the monitored liquid (m^2^·s^−1^); *l* the surface length in the flow direction, which generally corresponds to the length of the base of the wind tunnel (m); Re and Sc the Reynolds and Schmidt numbers, respectively (both dimensionless).

More specifically, the Reynolds and Schmidt numbers are dimensionless groups whose function it is to characterize convective mass transfer:

(8) $$K_{C}=\frac{0,664D_{i}}{l}{\left(\frac{v\rho l}{\mu }\right)}^{1/2}{\left(\frac{\mu }{D_{i}\rho }\right)}^{1/3}=0,664{\left(\frac{{D_{i}}^{4}\rho }{l^{3}\mu }\right)}^{1/6}v^{1/2}$$

where *v* is the average air velocity (m·s^−1^); ·*ρ* the air density (kg·m^−3^) and *μ* the air viscosity (kg·m^−1^·s^−1^).

Density and viscosity are functions of temperature; *v* is the velocity responsible for convective mass transfer, *i.e.*, the average velocity on the emitting surface.

By using Fick's Law to describe the mass transfer of a compound into a gas stream due to diffusion at the interface between liquid and gas phase, and by considering the material balance of compound *i*, assuming that the molar flow of compound *i* at the wind tunnel outlet is equal to the molar flow of *i* emitted at the liquid-gas interface, it is possible to obtain an equation that relates the concentration of the generic compound *i* to the transfer coefficient and gas stream velocity:

(9) $$c_{i}\propto \frac{K_{C}}{v}$$

According to the above-described Prandtl boundary layer theory, *K_C_* is proportional to *v*^1/2^. For this reason, in the case of convective mass transfer from a liquid to a gas phase, we can say that:

(10) $$c_{i}\propto v^{-1/2}$$

Analogously, if odor is considered to be:

(11) $$c_{od}\propto v^{-1/2}$$

given the relationship between odor concentration, SOER and OER, we can derive that [60,65]:

(11) $$\text{SOER},\text{OER}\propto v^{-1/2}$$

Based on this model, it is clear that odor concentration as well as SOER and OER is a function of air velocity on the sampled surface. In other words, environmental odor emissions from area sources depend on the wind speed on the emitting surface. Consequently, during sampling, odor emissions are a function of the air velocity inside the sampling hood.

This model is extremely useful when the results of olfactometric analyses obtained at specific sampling conditions (*i.e.*, air velocity inside the sampling hood) are used to refer to other conditions, for instance in real environmental conditions (*i.e.*, wind speed over the sampled surface), as it is the case for odor dispersion modeling purposes, whereby OERs from area sources have to be re-calculated for each hour of the simulation domain based on current wind speed [65]. Knowledge of the wind profile on the odorous surface is of great importance in such cases.

Certain scientific studies have also proved the effectiveness of the above-described model (Equations (10) and (11)) for liquid surface sources, demonstrating good correspondence between theoretical and experimental results [62].

This was not the case, however, for solid surface sources. This may be dependent on the fact that mass transfer from a solid to a gas phase is a more complex process, involving other phenomena, such as diffusion within the solid and effects of turbulence on the monitored surface. For this reason, other variables should probably be taken into account in the development of a suitable volatilization model, such as solid porosity and composition, surface roughness, *etc.* Nonetheless, surface-to-atmosphere volatilization of organic compounds may be described using models similar to those used for liquid surfaces [66]. It is also possible to express the SOER and the OER as an exponential function of the air velocity:

(12) $$\text{SOER},\text{OER}\propto v^{n}$$

where *n* is an experimental exponent that depends on the conditions under consideration and is generally not equal to 0.5, as is the case for liquids.

As previously mentioned in brief, the odor concentration measured at the wind tunnel outlet decreases as air velocity increases, as described by the above-reported Equations (9) and (10). In fact, it is difficult to measure odor concentration values below 50–100 ou_E_/m^3^ by dynamic olfactometry, as they tend to be similar to the typical values of non-odorous ambient air. For this reason, sampling on surfaces that are not highly emissive (e.g., wastewater oxidation tanks, exhausted landfill surfaces) may be problematic. In such cases, it is good practice to carry out sampling operations using low air flows, in order to have low air velocities on the surface to be sampled (about 1–10 cm/s), thereby preventing the odor concentration values at the hood outlet being too low to be measured [61,62]. Given the direct dependence of measured odor concentration on air velocity on the sampled surface, it is important to indicate the velocity adopted for odor sampling in the olfactometric report. Another important consideration regards odor dispersion models which should account for this dependence and thus re-calculate the OER for each hour of the simulation domain according to actual wind speed.

## 6. Conclusions and Future Trends

Sampling is a key issue in odor characterization and measurement, and may affect measureement quality to an even greater extent than the accuracy of the chosen measurement technique. The importance of this topic is evidenced by the large number of research groups working on the subject, as well as by recent specific regulations as a result of the publication of the German VDI 3880 guideline on odor sampling [26]. This German guideline will probably soon become the precursor for the draft of a European Norm, as happened in the past with VDI 3881 [25] on olfactometry, which became EN 13725:2003 [12], and with VDI 3940 on field inspections [67], which was discussed by a European Working Group (WG27) over the last few years and will soon be published as an EN [68].

This paper provides a complete overview of state-of-the art odor sampling for olfactometric analyses, and in doing so discusses recent standardizations as well as problematic aspects still under study.

Important odor sampling topics currently being researched or likely to be so in the near future [69] include, for instance, the improvement of the understanding and description of the physical and chemical phenomena responsible for odor emission which would help develop suitable models for emission rate estimation, particularly with regard to area sources [70,71], as well as diffusion mechanisms through sampling materials, which would improve sample conservation between sampling and analysis [72,73].
